# Community Turnover of Wood-Inhabiting Fungi across Hierarchical Spatial Scales

**DOI:** 10.1371/journal.pone.0103416

**Published:** 2014-07-24

**Authors:** Nerea Abrego, Gonzalo García-Baquero, Panu Halme, Otso Ovaskainen, Isabel Salcedo

**Affiliations:** 1 Department of Biological and Environmental Science, University of Jyväskylä, Jyväskylä, Finland; 2 Department of Plant Biology and Ecology, University of the Basque Country (UPV/EHU), Bilbao, Spain; 3 Natural History Museum, University of Jyväskylä, Jyväskylä, Finland; 4 Department of Biosciences, University of Helsinki, Helsinki, Finland; Institut Pasteur, France

## Abstract

For efficient use of conservation resources it is important to determine how species diversity changes across spatial scales. In many poorly known species groups little is known about at which spatial scales the conservation efforts should be focused. Here we examined how the community turnover of wood-inhabiting fungi is realised at three hierarchical levels, and how much of community variation is explained by variation in resource composition and spatial proximity. The hierarchical study design consisted of management type (fixed factor), forest site (random factor, nested within management type) and study plots (randomly placed plots within each study site). To examine how species richness varied across the three hierarchical scales, randomized species accumulation curves and additive partitioning of species richness were applied. To analyse variation in wood-inhabiting species and dead wood composition at each scale, linear and Permanova modelling approaches were used. Wood-inhabiting fungal communities were dominated by rare and infrequent species. The similarity of fungal communities was higher within sites and within management categories than among sites or between the two management categories, and it decreased with increasing distance among the sampling plots and with decreasing similarity of dead wood resources. However, only a small part of community variation could be explained by these factors. The species present in managed forests were in a large extent a subset of those species present in natural forests. Our results suggest that in particular the protection of rare species requires a large total area. As managed forests have only little additional value complementing the diversity of natural forests, the conservation of natural forests is the key to ecologically effective conservation. As the dissimilarity of fungal communities increases with distance, the conserved natural forest sites should be broadly distributed in space, yet the individual conserved areas should be large enough to ensure local persistence.

## Introduction

Biodiversity is decreasing faster than ever in the history of the earth, largely as a result of human activity [1]. Consequently, conservation and management actions aiming at sustainable use of ecosystems and maintenance of biological diversity have become a priority worldwide. The most commonly applied conservation measures in terrestrial ecosystems have focused on preserving key habitats, species or communities of conservation interest. To date, most conservation strategies have focused on designating protected set aside areas or increasing the areas of existing protected areas. For example, regarding temperate forests, a considerable proportion of the remaining old-growth and semi-natural forests are already conserved, and the protected area is expected to continue increasing [2]. To make conservation efforts cost-effective in an evidence based manner, protection efforts need to be supported by research about the effects of habitat protection [2]. One factor potentially compromising the cost-effectiveness of biodiversity conservation plans is that the patterns and drivers of distribution and turnover of biodiversity across different spatial scales are still poorly understood for many organism groups [3].

A successful design of conservation area networks requires an understanding of the processes determining species composition and persistence at different spatial and temporal scales [4]. For example, if local processes are the most important factor determining the persistence and diversity of the focal species group (as has been observed for several taxa; [5]–[7]), it may be more effective to aim management actions or site selection procedures towards maintaining the heterogeneity and variability of habitats at the local scale, instead of e.g. increasing the connectivity among protected areas [5]. The influences of local versus regional processes in community assembly often interact with the traits of the species. For example, in a study of forest-dwelling beetles, Gering et al. [8] found that the spatial turnover of rare species was realised across large spatial scales (ecoregions), whereas the spatial turnover of common species was realised across smaller spatial scales. The most commonly used concept in measuring spatial species turnover is beta-diversity. However, this concept has been defined and used in multiple ways, making it difficult to synthesize studies based on this concept, and leading to a debate on its correct use [9], [10].

Wood-inhabiting fungi are a diverse and functionally important group of organisms in forest ecosystems. In addition to their central role in nutrient cycling [11], they play a key role on the creation of new habitats and resources for other wood-dwelling species [12]. Furthermore, their dependence on dead wood makes them especially vulnerable to the changes caused by forest management (e.g. [13], [14]). At the resource unit level, most species show preferences to a specific substrate type in terms of host species, log type (snag, log, stump…), decay stage and log size [15]. Therefore, the reduction of particular dead wood types (especially of coarse woody debris) in managed forests has directly led to declines and extinctions of certain species [16], [17]. At the stand level, fungal communities are highly influenced by microclimatic conditions, which modify e.g. the water content of dead wood and thus can inhibit or facilitate the colonization and fructification of species [18], [19]. Further, the size and isolation of forest stands can significantly influence fungal communities: specialist species have been found to be negatively affected by isolation while generalist species can even benefit from fragmented surroundings [15]. At the broadest scale, wood-inhabiting fungi are influenced by regional factors, related e.g. to climatic variation and management history [20].

Only a few studies have examined the importance of environmental factors at different spatial scales in determining the diversity of wood-inhabiting fungi [21], [22]. Berglund et al. [23] applied a hierarchical study design and a hierarchical data analysis to show that habitat suitability determines the presence of polypore species at a small spatial scale. Bässler et al. [24] quantified the relative importance of the plot and forest management levels, showing that in spruce forests, species turnover is higher among plots than among management categories. Based on these previous studies, wood-inhabiting fungal species diversity is much affected by small scale processes, though also regional-scale processes seem important especially for specialized and threatened species. However, we are not aware of any previous studies in which the variation in species richness and community composition have been explicitly partitioned to different spatial scales. Such information would be critically needed to design effective conservation areas.

The overall objective of this study is to partition the variation in the assembly of wood-inhabiting fungal communities into different spatial levels. More specifically, our aim is to examine how community turnover of wood-inhabiting fungi is realised at the levels of forest management types, forest sites and plots, and how much of community variability is explained by variation in resource composition and spatial proximity. We utilize the conclusions of the present study to derive recommendations for the design of protection area networks for the conservation of wood-inhabiting fungi in temperate broadleaved forests.

## Materials and Methods

Study permits were provided by the Government of Navarre (the local official institution for forest management) and the field studies did not involve endangered or protected species.

### 1. Study area

The study was carried out in the northern part of Navarre in northern Spain. The study area covers two biogeographical regions with temperate climate: the Atlantic region in the Northwest and the Alpine region in the Northeast. The predominant forest type is beech-dominated forest (with total area of ca. 61,000 ha, covering ca. 14% of the landscape), which also represents the most productive forest type for commercial harvesting in the area.

The northern part of Navarre consists of a valley-dominated landscape with a mountainous topography. Towns and farms are generally located in the lower parts of the valleys and the forests grow in the hillsides. The major part of the forested area has been intensively managed by thinning and selective cutting, but there is also a network of permanently protected areas (see Files S1 and S2). In this study, we examined the difference between two management types, called henceforth managed and natural forests. As natural forests we considered such protected areas in which logging activities have been prohibited, allowing the development of old-growth beech forests. As managed forests we considered such unprotected sites in which commercial harvesting has been continuously conducted (File S1).

### 2. Study design

#### 2.1. Sampling design

We assembled a list of natural and managed forest sites based on the above definitions. From this list, eight managed and eight natural forest sites (File S1) were randomly selected. Within each site, fungal data (species abundances, defined as number of logs in which each species was observed) were recorded in five randomly placed 100 m^2^ sampling plots (File S2). The sampling plots were separated from each other by tens of meters to hundreds of meters, whereas the study sites were separated by hundreds of meters to kilometres (the minimum, maximum and mean distances between plots were 0.07, 114 and 44 km respectively). The sites belonging to the two management types were distributed in space without a strong spatial autocorrelation, i.e. the managed and natural sites did not form their own clusters. The nested design has thus two factors: management (considered as fixed factor; with two levels) and forest site (considered as random factor; with eight levels, nested within management). Variation among study sites represents spatial variation at a regional scale, whereas the residual variation among the sampling plots represents spatial variation at a local scale.

#### 2.2. Data collection

The fieldwork was carried out in the year 2011 during the main period of fungal fruit body production (from late September to early November). All woody debris (called henceforth resource units) with minimum diameter of 1 cm were measured for diameter and decay class, and examined for the presence of fungal species. The resource units were classified according to their diameter (three classes) and decay stage (five classes), thus forming altogether 15 classes. The diameter classes correspond to those used by Abrego & Salcedo [17]: very fine woody debris (VFWD), including resource units with diameter in the range 1–5 cm; fine woody debris (FWD), with 5 cm<diameter ≤10 cm; and coarse woody debris (CWD), with diameter >10 cm. The decay stages were classified into 5 categories based on a modified version of the classification by Renvall [25].

The presence of all saproxylic macromycetes (fungi with visible fruit bodies) was registered at the level of the resource units. Microscopic identification was carried out in the laboratory when necessary. The literature consulted for identifying fungal fruit bodies is summarized in File S3. Each species found on one resource unit was considered as one record, regardless of the number of fruit bodies. Thus, the abundances of the species correspond to the number of woody debris pieces in which each species was found in each sampling plot.

### 3. Statistical analyses

We first conducted a descriptive analysis examining how species richness in fungal communities varies across the three hierarchical scales included in this study. To do so, we constructed accumulation curves in which the total number of species was computed as a function of the number of surveyed plots, assuming that the plots were selected randomly either a) within the same randomly selected managed forest site, b) within the same randomly selected natural forest site, c) among all sites in managed forests, d) among all sites in natural forests, or e) among all sites. We estimated the mean number of species and the interquartile (25%–75%) range for each of these five cases and for each number of sampling plots using 1000 random replicates.

We supplemented the accumulation curves by an additive partitioning in species richness at different hierarchical scales [26], [27]. In this methodology, the total diversity *γ* (here, species richness) is modelled as a sum of the average diversities within sampling units (*α*) and among sampling units (*β*), the latter across the scales included in the hierarchical design. In our case, the partitioning of species richness *γ* was thus expressed as 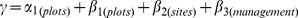, where *α*_1_ is the mean species number in the sampling plots, *β*_1_ is the turnover (beta-diversity) between sampling plots, *β*_2_ is the turnover between forest sites, and *β*_3_ is the turnover between managed and natural forests. The additive partitioning and the related significance tests were performed under 999 permutations of the null model r0 of the function *adipart* in the R-package *vegan* [28]. The null model r0 is suited to binary (presence/absence) data, and it randomizes the community matrix in a way that preserves the number of species observed in each site.

We then analysed variation in community composition at the different levels of our hierarchical study design by calculating Bray-Curtis similarity measures [29] for all pairs of sampling plots. We constructed similarity matrices both for the fungal data (square root transformed species abundances, organised as a matrix for 80 sampling plots by 285 species) and for the dead wood data (square root transformed counts of resource unit types, organised as a matrix of 80 sampling plots by 15 resource unit types). We examined how much of the similarity in dead wood composition (SDC) was explained by the two hierarchical factors of management and site, as well as spatial distance (D). We examined how much of the similarity in fungal community composition (SFC) was explained by management and site, as well as by similarity in dead wood composition (SDC) and spatial distance (D). To do so, we constructed matrices measuring similarity in site (SS; with value 1 if the plots belonged to the same site and 0 otherwise), similarity in management (SM; with value 0 if the plots belonged to a different management category, and values 1 or 2 if both plots belonged to the managed or to the natural category, respectively) and log-transformed spatial distance (D) between the sampling plots. We treated SS and SM as fixed factors and SDC and D as continuous covariates. In the models (SDC = SS+SM+D and SFC = SS+SM+SDC+D) we considered also all two-way interactions (except for D). We used backward variable selection to include in the final model only the statistically significant variables. To account for the non-independence of data points that relate to pairs of plots rather than to individual plots, we tested for the statistical significance by comparing the *F-values* related to each factor to the null distribution of *F-values.* To compute the null distribution, we constructed 999 permutations of the species community, in each of which we permuted randomly the identity numbers of the sampling plots. As a complementary approach to quantify the variation on species and resource similarity explained by each scale, we carried out a permutational multivariate analysis of variance (PERMANOVA; [30]). The *pseudo-F* values were used to evaluate which components of the models explained more variability than expected by a null model. This analysis was performed using 999 permutations of residuals under a reduced model with the function *nested.npmanova* of the R-package *BiodiversityR.*

All statistical analyses were performed using R software [31], the code and the original data (the fungal species data matrix, UTM locations of all sampling plots and resource type data matrix) being available in the Supporting Information section (Files S4–S7).

## Results

### 1. Variation in species richness and resource types

**Figure 1:**
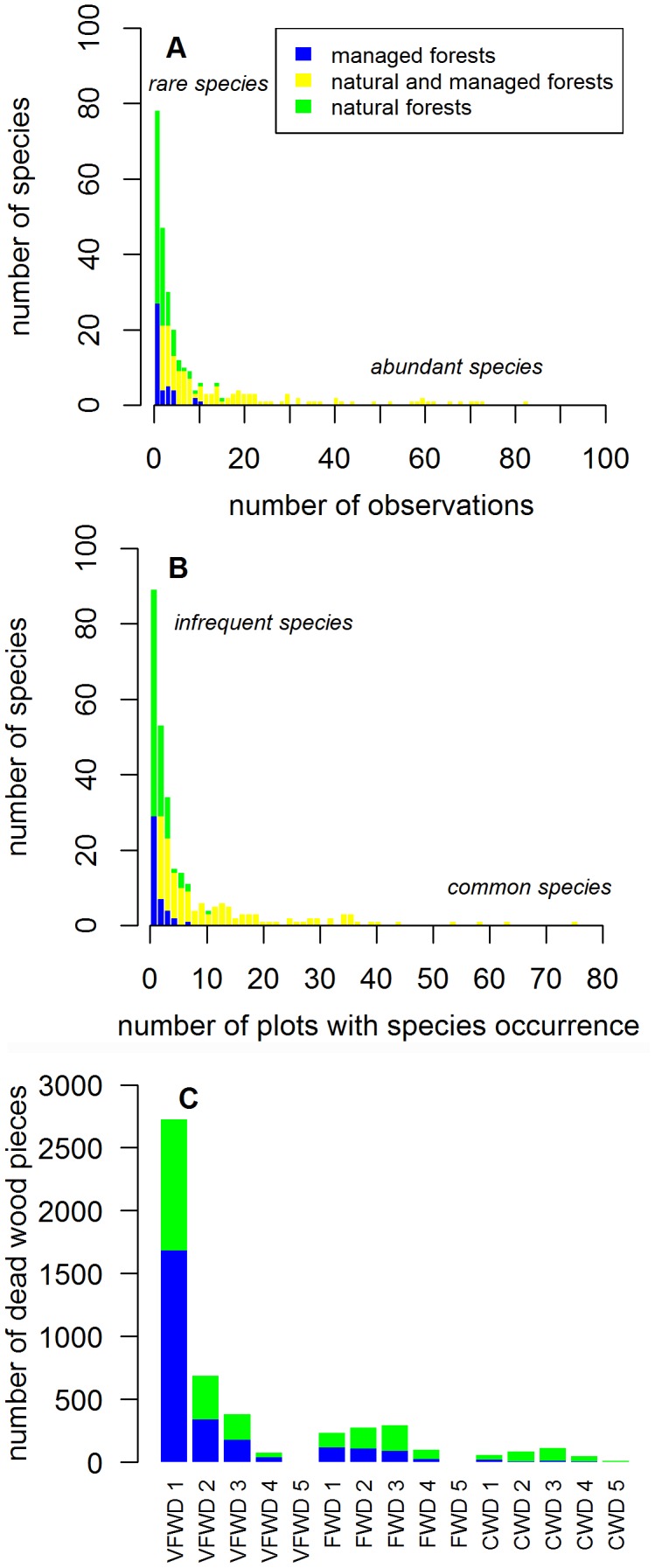
The abundance distributions of species and dead wood types. The total number of occurrences of each species (Panel A), the number of plots in which each species was recorded (Panel B) and the number of occurrences of each dead wood type (Panel C). The species and dead wood pieces that were recorded in managed forests are represented with blue colour, those that were recorded in natural forests with yellow colour, and those species recorded in both forest types with green colour.

We recorded in total 3241 occurrences of 285 species in 2724 dead wood pieces. The species abundance distribution was dominated by rare species (Fig. 1A), with 27% of the recorded species (2.4% of the occurrences) being represented only once in our data. The five most abundant species were simultaneously the five species with highest prevalence at the plot level, comprising over 25% of all occurrences. In general, the species with high abundances were found in both (managed and natural) forest types (Fig. 1, A and B; black bars). Nevertheless, note that in the tail of Fig. 1 (A) there are also some species that were found only in managed forests. These species are *Steccherinum fimbriatum* and *Hyphodermella rosae*, which were recorded in 7 managed plots, 18 and 11 times respectively.

The dead wood profile was numerically strongly dominated by small diameter classes in early decay stages (Fig. 1C). The coarser dead wood classes in the latest decay stages were the most uncommon, and were mostly found in natural forests.

**Figure 2:**
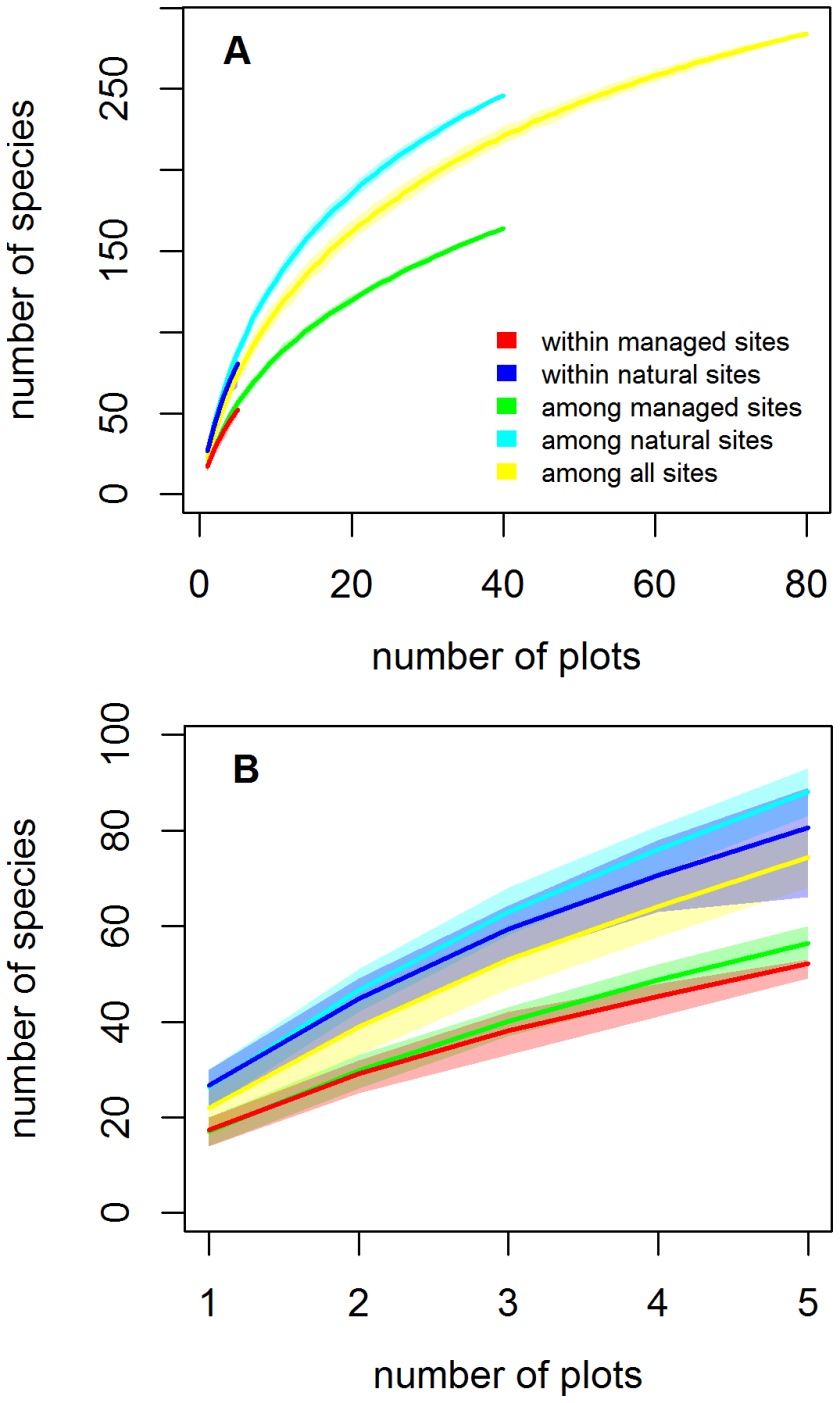
Species accumulation curves showing the total number of species in a given number of plots. The thick lines show means and the shaded regions the interquartile (25%–75%) ranges. The different lines correspond to the five cases which assume that the plots were selected randomly either within the same randomly selected managed forest site (red), the same randomly selected natural forest site (dark blue), among all sites in managed forests (green), among all sites in natural forests (light blue), or among all sites (yellow). Note that the maximum number of plots for which the number of species could be computed is either 5, 40 or 80, depending on the category. Panel A shows the full data, panel B a zoom, in which the number of plots varies from 1 to 5.

The species accumulation curves indicate that natural forests were substantially more species rich than managed forests (Fig. 2A). Further, the number of species increased much faster if the sampling plots were located only in natural forests (Fig. 2A; light blue line) than if they were located both in natural and managed forests (Fig. 2A; yellow line), suggesting that the species assemblages in managed forests were largely a subset of those present in natural forests. The total number of species increased only marginally faster if the sampling plots were spread across forest sites than if they were located within a forest site, both in managed (Fig. 2B; green and red lines) and natural (Fig. 2B; light blue and dark blue lines).

**Figure 3:**
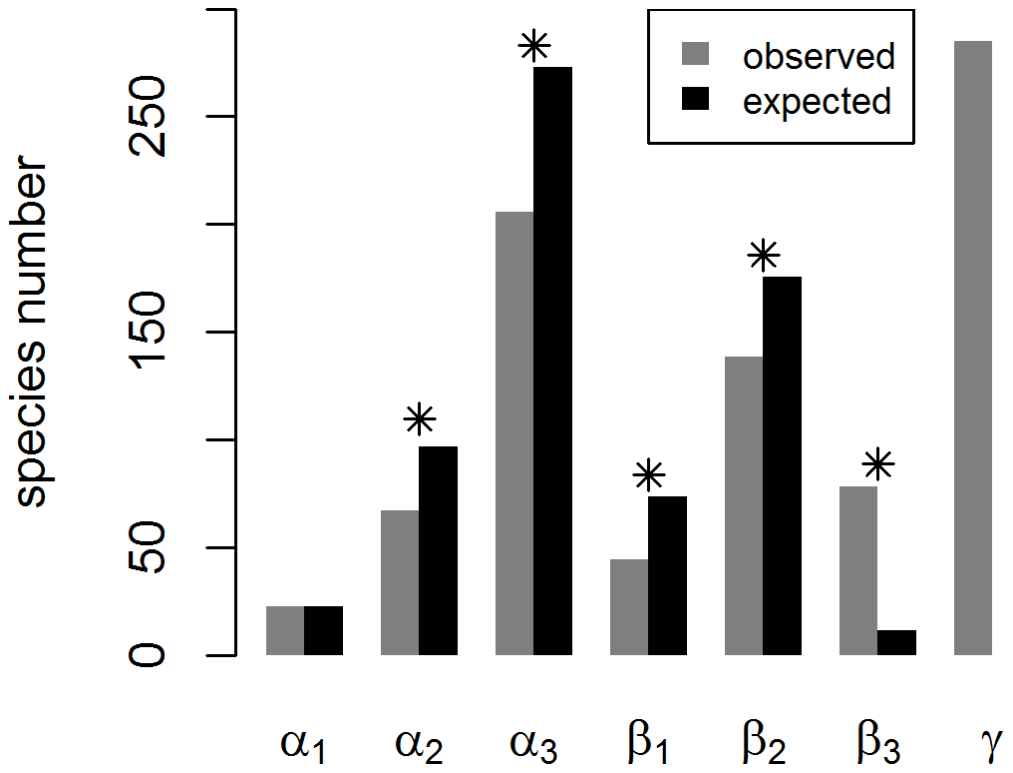
Total species richness (γ) explained by alpha and beta components of diversity at three spatial scales: the mean species number per plot (α_1_), the mean species number per site (α_2_), the mean species number per management category (α_3_), the average addition of species from plots to sites (β_1_), the average addition of species from sites to management categories (β_2_), the average addition of species from management categories to the total species number (β_3_) and the total species number (γ). The grey bars correspond to the observed values and the black bars to the expected values based on a null model. The asterisks indicate the levels of statistical significance (* corresponding to *p*<0.001) for a difference between observed and expected values.

The additive partitioning of total species richness (Fig. 3) was in line with the patterns visible in the species accumulation curves. The increase in species number when moving from the plot level to the forest site level (β_1_) was lower than would expected by chance, and consequently so was also the number of species within a site, averaged across the sites (α_2_). Similarly, the increase in species number when moving from the site level to the management level (β_2_) was lower than would expected by chance, and consequently so was also the mean number of species within a management category, averaged across the two management categories (α_3_). Both of these results reflect the fact that the most important part of species diversity was realized among the management categories (β_3_). This is consistent with Fig. 2, which shows that natural forests were much more species rich than management forests, and that managed forests largely contained a subset of the species found from natural forests. Note that α_3_ corresponds to the mean of the light blue and green lines in Fig. 2, which indeed is below the yellow line, which corresponds to a null expectation.

### 2. Variation in community and resource similarities

**Figure 4:**
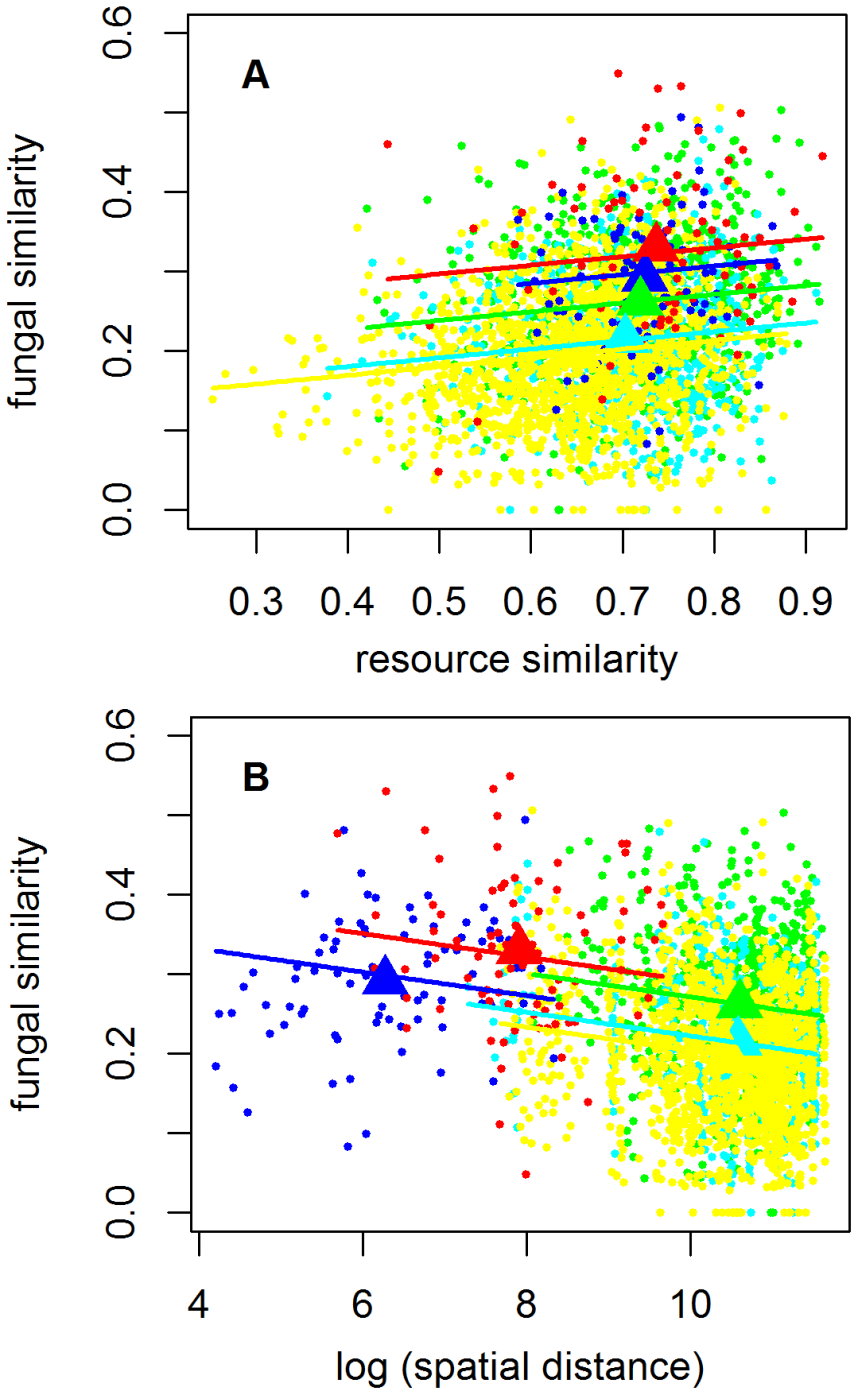
Predicted similarity of the fungal composition (SFC), among sampling plots. When two sampling plots are located either within the same managed forest site are represented by red colour, when located within the same natural forest site are represented by dark blue colour, when located in two different sites in managed forests are represented by green colour, when located in two different sites in natural forests are represented by light blue colour, and when plots are located in sites from different management types are represented by yellow colour. The dots show raw data, the triangles the mean values and the lines the model predictions. When constructing the model prediction as a function of resource similarity (panel A), we normalized spatial distance to its mean value within each category. When constructing the model prediction as a function of spatial distance (panel B), we normalized resource similarity to its mean value within each category.

After applying the model selection procedure, the model for similarity in fungal community composition (SFC) included as explanatory variables management type similarity (SM), forest site similarity (SS), similarity in dead wood composition (SDC) and spatial distance (D) (Table 1). The model for similarity in dead wood composition (SDC) included management type similarity (SM) and forest site similarity (SS) but not spatial distance (Table 1). In both models none of the two-way interactions were significant (Permutation comparison of *F-values, p>*0.05). The variability explained by both models was low, as only the 17% of the total variability was explained in the SFC model and 11% in the SDC model. According to the predicted values of these models, the plots within managed forests showed higher fungal and dead wood similarities than the plots within natural forests, and the lowest fungal and dead wood similarities were found among pairs of plots out which one belonged to a natural and the other one to a managed forest (Fig. 4A). The effect of forest site similarity (SS) on the similarity of both fungal composition (SFC) and dead wood was small but yet significant (Fig. 4, Table 1). Fungal community similarity decreased with increasing spatial distance (Fig. 4B).

**Table 1:** Linear models for similarity in fungal and dead wood composition.

*Response variable: Similarity in fungal composition (SFC)*						
Source of variation	df	Estimate	SSq	MS	F	p
Management(SM)	2	MM: 0MU: −0.060UU: −0.047	1.93	0.96	152	<0.001
Site(SS)	1	Different: 0Same: 0.019	0.71	0.71	112	<0.001
Similarity in dead wood composition(SDC)	1	0.11	1.11	1.11	175	<0.001
Spatial distance(D)	1	−0.02	0.46	0.46	73	<0.001
Residual	3154		20.02	0.01		
*Response variable: Similarity in dead wood composition (SDC)*						
Source of variation	df	Estimate	SSq	MS	F	p
Management(SM)	2	MM: 0MU: −0.067UU: −0.016	3.07	1.54	192	<0.001
Site(SS)	1	Different: 0Same: 0.019	0.05	0.05	6.58	<0.001
Residual	3156		25.26	0.01		

df = degrees of freedom, SSq = sum of squares, MS = mean squares, MM = pairs of plots from managed forests, MU = pairs of plots belonging to a different management types, UU = pairs of plots from natural (unmanaged) forests. Statistical significance (*p*) based on permutation test.

The results from the Permanova analysis (Table 2) were in line with the results of the linear model described above: for both fungal and dead wood species composition, both forest management type and forest site level explained a significant amount of variation. For dead wood composition, the variation explained by forest management type was much higher than that explained by forest site level.

**Table 2:** Output from the Permanova analysis for fungal species and dead wood composition models.

		*Response variable: Similarity in fungal composition (SFC)*				*Response variable: Similarity in dead wood composition (SDC)*			
Source of variation	df	SS	MS	*Pseudo-F*	*p*	SSq	MS	*Pseudo-F*	*p*
Management	1	17737	17737	3.53	0.001	8549.2	8549.2	12.45	0.001
Site	14	70343	5024.5	2.08	0.001	9612.1	686.58	1.74	0.002
Residual	64	154670	2416.7			25288	395.13		

df = degrees of freedom, SSq = sum of squares, MS = mean squares.

## Discussion

We found that the assembly of fungal communities was influenced by all scales that we considered in this study. Indeed, the similarity of fungal communities was higher within sites and within management categories than among sites or between the two management categories; it decreased with increasing distance among the sampling plots and increased with increasing similarity of dead wood resources. However, the majority of the variation remained unexplained, suggesting that biological interactions, neutral mechanisms such as inherent randomness in dispersal, establishment and fruiting [32], as well as environmental constraints not considered here are likely to play an important role in determining local assemblies of wood-inhabiting fungi. This result partly reflects the fact that the wood-inhabiting fungal communities studied here were dominated by locally rare and infrequent species, which directly leads to a high species turnover from one plot to another. This result is in line with a previous study in a coniferous forest where a high variability of wood inhabiting fungal species at small scales was also detected [24].

Bässler et al. [24] partitioned wood-inhabiting fungal richness between plots and management categories, with the result that community turnover was higher between study plots than between management categories. Thus Bässler et al. [24] concluded that the stand level is the most relevant scale for the conservation the wood-inhabiting diversity. In contrast, we found that fungal communities were significantly influenced by all scales that we considered, and in particular by the management category, as natural forests were significantly more diverse than managed forests. The difference between our results and those of Bässler et al. [24] can be at least partially attributed to a difference in the study design, as the study of Bässler et al. [24] involved only a single forest site for each management category.

In general, wood-inhabiting fungal species show preferences for certain resource types in terms of tree species, size and decay class [15], so from a niche differentiation perspective [33], variation in resource type is expected to explain a major part of species variation at a small scale. In our study, although increasing similarity in resource composition (as measured by dead wood decay class and size) significantly increased the similarity of wood-inhabiting fungal communities, the trend was weak. Hence, at a small spatial scale, wood-inhabiting fungal communities seem to be largely influenced by stochastic processes related to species’ idiosyncrasy and spatial proximity, rather than habitat factors. Some hierarchically designed studies have reported similar results, and showed that the variation in the occurrence of species among different forest stands [23] or study plots [34] remains largely unexplained even when the most important explanatory variables were considered.

The dispersal capabilities of species can be considered as a species-specific factor limiting potentially their occurrence on suitable habitats [35], [36]. One of the covariates that significantly influenced fungal species similarity in our study was the spatial distance, with the result that similarity in fungal composition decreased with increasing distance between the study plots. This result suggests that dispersal limitation is one major factor shaping the local assembly of fungal communities.

Other factors creating unexplained variation between study units are related to the low detectability of wood-inhabiting fungi [37], partly due to their fruiting phenology and its dependence on microclimatic variation [38]. Many fungal species have ephemeral, short living fruit bodies, which makes it difficult to assess fungal diversity by a single fruit body based survey [37]. Further, a large proportion of fungal species can never be detected as fruit bodies [39]. With presence-absence data, low detectability generates a larger amount of random variation on small spatial scales because on larger scale the increasing number of potentially detectable individuals increases the likelihood that at least one is detected [40]. Thus, also in our study, the low general detectability of fungi as well as the climatic dependence of their phenology may have inflated the variation realized at the smallest spatial scale.

Due to the overall high number of species and the large proportion of rare species, the species accumulation curves we constructed were far from saturated. The only way to achieve saturated accumulation curves for hyperdiverse organism groups consists of sampling intensively over a limited area during several years [8], [41]. Nevertheless, in spatially extensive studies even unsaturated accumulation curves provide a suitable method for the assessment of the heterogeneity of species diversity [8].

It is well documented that wood-inhabiting fungal communities in natural forests are significantly more species rich and different from those in managed forests (e.g. [16], [42], [43]), including the communities in beech forests [17]. The main factors behind this difference are related to resource availability and connectivity at forest stand level [15], [42]. Our previous study [17] showed that species specialised in utilizing certain dead wood types were absent from managed forests, due to the absence of suitable resource type. Based on the previous [17] and present results, the community differences between different management categories are not related to qualitative differences in species compositions, but to quantitative differences, in the sense that the species from managed forests are a subset from those in natural ones (because their required resource has disappeared). Therefore we cannot say that the natural and managed forests have different species compositions, but rather that natural forests have more species. Indeed, the general trend reported in the literature is that the reduction of particular dead wood pieces caused by forestry practices in managed forests causes extinctions of habitat specialist saproxylic species (e.g. [44]–[46]). Thus, forest management significantly affects resource composition, and managed forests are more homogeneous in their dead wood and fungal species compositions. Nevertheless we found some species to be more abundant in managed forests than in natural ones, in particular *Steccherinum fimbriatum* and *Hyphodermella rosae.* Consistently with our results, earlier studies have shown that there are species adapted to disturbances, such as those caused by forest management [15], [47].

### Conservation implications and conclusions

Identifying the spatial scales at which species vary in their diversity and composition is an important research goal in conservation ecology [4], [48]. Additive partitioning of diversity is one of the most commonly applied statistical approaches for quantifying the fraction of diversity explained by different scales and for concluding which scales are critical for species conservation (e.g. [5]–[8]). We found that fungal communities show a high level of turnover at all the studied scales, and that only a small part of the turnover can be explained by factors such as spatial proximity or resource type similarity. The large number of random variation and high number of rare species suggests that a large total area of protected forests is needed to conserve a substantial part of fungal diversity. As managed forests contributed only little to the total number of species, an ecologically effective way to conserve fungal communities necessarily requires conserving natural forests. Nevertheless, leaving dead wood in managed forests can be seen an additional supplementary method, partly because some rare species only appear in managed forests, and partly because increasing the quality of the matrix (here, managed forests) can be essential for ensuring the long-term viability of populations in fragmented landscapes [49]. We found the similarity of fungal communities to decrease with increasing spatial distance, suggesting that protected areas should be distributed thorough the landscape. However, the individual protected areas or local networks of protected areas must simultaneously be kept large enough to ensure the persistence of local populations, which process we did not however consider in the present study.

## Supporting Information

File S1 Principal characteristics of the sampled beech forest sites.
(DOC)

File S2 A map of the sampled forest sites and plots in Navarre.
(TIF)

File S3 Literature consulted for the identification of wood-inhabiting species.
(DOC)

File S4 Code for performing the analyses in R.
(TXT)

File S5 Fungal species data.
(CSV)

File S6 Geographical UTM coordinates.
(CSV)

File S7 Dead wood composition data.
(CSV)
