# Supplementary material for: Community Turnover of Wood-Inhabiting Fungi across Hierarchical Spatial Scales
Source: PLoS One. 2014 Jul 24;9(7):e103416. doi: 10.1371/journal.pone.0103416 (PMC4110023; doi:10.1371/journal.pone.0103416)
Supplement: File S1 — Principal characteristics of the sampled beech forest sites. (DOC) [file pone.0103416.s001.doc]

**Appendix A**. Principal characteristics of the sampled beech forest sites.

| **CONSERVATION AREA** | **CONSERVATION STATUS** | **BIOGEOGRAPHICAL REGION** | **SITE AREA**  **(ha)** | **PRIORITARY HABITATS %** | **ALTITUDE**  **(m)** | **PRECIPITATION (mm)** | **MEAN**  **TEMPERATURE**  **(ºC)** | **BEECH AREA**  **(ha)** | **CONSIDERED FOREST TYPE** | **MANAGEMENT HISTORY** | **BEECH FOREST TYPE** |
| --- | --- | --- | --- | --- | --- | --- | --- | --- | --- | --- | --- |
| **Artikutza** | SCI | Atlantic | 3646 | 67% | 250-1054 | 2700 | 12 | 2550 | Natural | Last partial forestry intervention in 1919 (old growth forest) | Cantabric acidophilous |
| **Aztaparreta** | IR | Alpine | 175 | 100% | 1212-1726 | 1800 | 7 | 100 | Natural | Most of the area never managed (virgin forest) | Pyrenean |
| **Bertiz** | SCI - NP | Atlantic | 2052 | 96% | 175-816 | 1600 | 13 | 1764 | Natural | Last partial forestry intervention in 1949 and formations that have remained over 300 or 400 years present (old growth forest) | Cantabric acidophilous |
| **Irubetakaskoa** | NR | Atlantic | 116 | 100% | 199-668 | 2200 | 13 | 71 | Natural | Most of the area without forestry intervention at least in the last century (old growth forest) | Cantabric acidophilous |
| **Larra** | NR | Alpine | 2731 | 90% | 1083-2438 | 1800 | 7 | 471 | Natural | Not forestry intervention in the beech forest area at least in the last century (old growth forest) | Pyrenean |
| **Lizardoia** | IR | Alpine | 64 | 100% | 904-1183 | 1800 | 8 | 64 | Natural | Most of the area without forestry intervention at least in the last century (old growth forest) | Pyrenean |
| **Mendilatz** | NR | Alpine | 140 | 95% | 1030-1249 | 2000 | 8 | 140 | Natural | Not forestry intervention at least in the last century (old growth forest) | Pyrenean |
| **Putxerri** | NR | Atlantic | 83 | 100% | 1026-1291 | 1400-1500 | 11 | 83 | Natural | Not forestry intervention at least in the last century (old growth forest) | Cantabric basophilous |
| **Aldude** | SAC | Atlantic | 9024 | 86% | 590-1459 | 2000 | 10 | 4515 | Managed | Coal and clear-cuts (until the mid 20th century) and thinning today | Cantabric acidophilous |
| **Aralar** | SCI | Atlantic | 14026 | 37% | 460-1427 | 1400-1500 | 11 | 2100 | Managed | Coal (until the mid 20th century) and thinning today | Cantabric basophilous |
| **Aritzakun-Urritzate** | SCI | Atlantic | 5822 | 67% | 380-1072 | 2200 | 13 | 1282 | Managed | Coal and clear-cuts (until the mid 20th century) and thinning today | Cantabric-acidophilous |
| **Belate** | SCI | Atlantic | 24830 | 52% | 180-1418 | 1400-1500 | 10 | 6143 | Managed | Coal and clear-cuts (until the mid 20th century) and thinning today | Cantabric acidophilous |
| **Larrondo-Lakartxela** | SAC | Alpine | 2152 | 68% | 1600-1844 | 1800 | 7 | 496 | Managed | Coal (until the mid 20th century) and thinning today | Pyrenean |
| **Orreaga-Irati** | SAC | Atlantic and Alpine | 17039 | 54% | 910-2018 | 1800 | 8 | 9600 | Managed | Coal (until the mid 20th century) and thinning today | Cantabric acidophilous and Pyrenean |
| **Otsagi** | None | Alpine | 11500 | 25% | 750-1000 | 1300-1400 | 10 | 11500 | Managed | Coal (until the mid 20th century) and some clear-cuts and thinning today | Pyrenean |
| **Urbasa-Andia** | Natural Park - SAC | Atlantic | 25398 | 48% | 560-1494 | 1300-1400 | 10 | 3900 | Managed | Coal (until the mid 20th century) and thinning today | Cantabric basophilous |

Abbreviations: IR=Integral Reserve, NP=Natural Park, NR=Natural Reserve, SAC= Special Areas of Conservation, SCI= Sites of Community Importance.
