# Supplementary figures and images for: Community Turnover of Wood-Inhabiting Fungi across Hierarchical Spatial Scales
Source: PLoS One. 2014 Jul 24;9(7):e103416. doi: 10.1371/journal.pone.0103416 (PMC4110023; doi:10.1371/journal.pone.0103416)

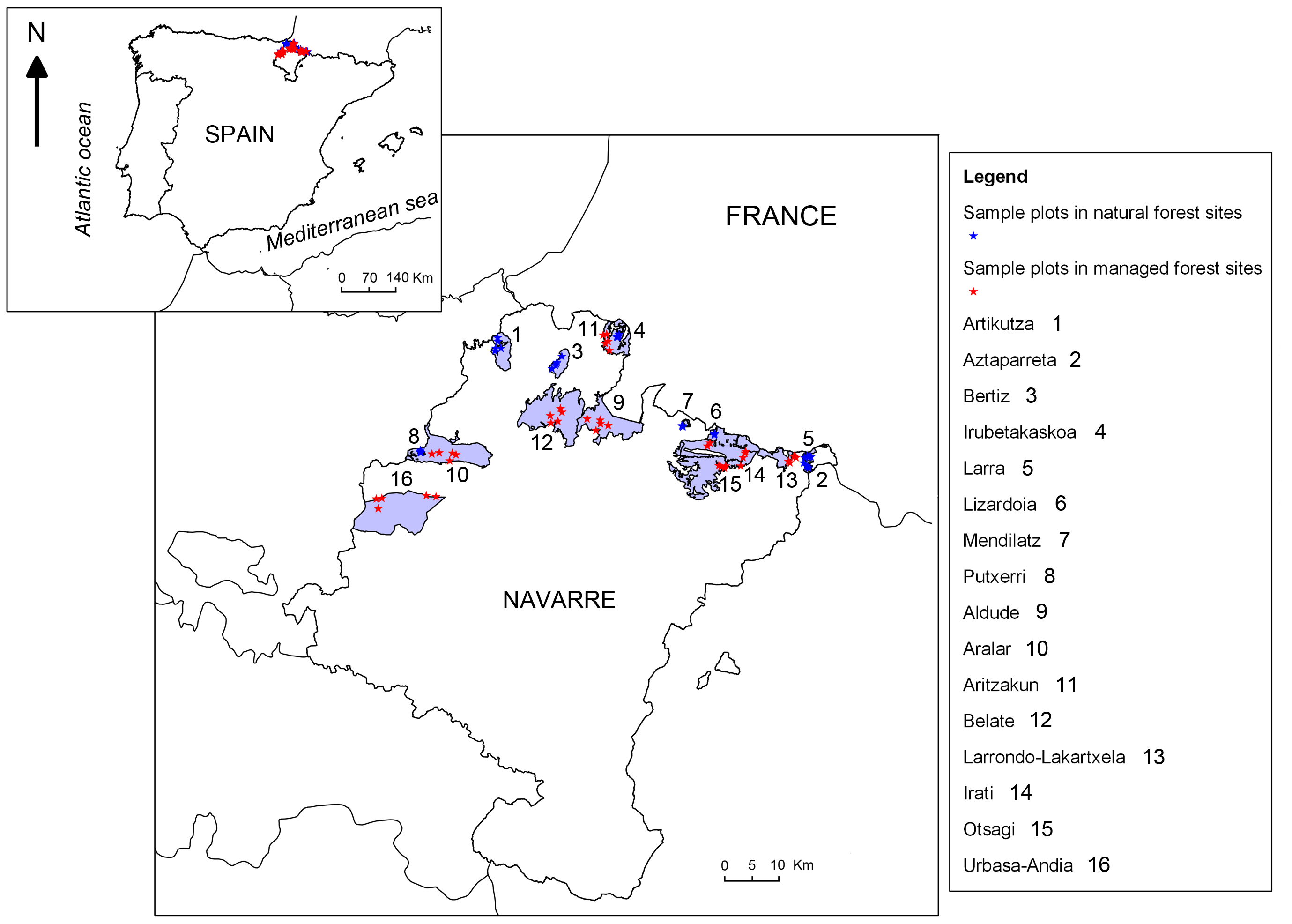

Supplement: File S2 — A map of the sampled forest sites and plots in Navarre. (TIF) [file pone.0103416.s002.tif]
