# Supplementary material for: Community Turnover of Wood-Inhabiting Fungi across Hierarchical Spatial Scales
Source: PLoS One. 2014 Jul 24;9(7):e103416. doi: 10.1371/journal.pone.0103416 (PMC4110023; doi:10.1371/journal.pone.0103416)
Supplement: File S3 — Literature consulted for the identification of wood-inhabiting species. (DOC) [file pone.0103416.s003.doc]

1. Bernicchia A. (2005) Fungi europaei: *Polyporaceae* s.l. Alassio, Italy: Edizioni Candusso.

2. Bernicchia A, Gorjón SP. (2010) Fungi Europaei: *Corticiaceae* s.l. Alassio, Italy: Fungi Europaei nº 12. Edizioni Candusso.

3. Breitenbach J, Kranzlin F. (1986) Fungi of Switzerland II (Heterobasidiomycetes, Aphyllophorales, Gastromycetes). Luzern, Switzerland: Mykologia Luzern.

4. Breitenbach J, Kränzlin F. (1995) Fungi of Switzerland, IV (Boletes and Agarics [part 2]). Luzern, Switzerland: Mykologia Luzern.

5. Breitenbach J, Kränzlin F. (1991) Fungi of Switzerland, III (Boletes and Agarics [part 1]). Luzern, Switzerland: Mykologia Luzern.

6. Breitenbach J, Kränzlin F. (1984) Fungi of Switzerland I (Ascomycetes). Luzern, Switzerland: Mykologia Luzern.

7. Eriksson J, Hjortstam K, Ryvarden L. (1984) The Corticiaceae of North Europe (*Schizopora- Suillosporium*). Oslo, Norway: Fungiflora. 1279-1449 p.

8. Eriksson J, Hjortstam K, Ryvarden L. (1981) The Corticiaceae of North Europe (*Phlebia- Sarcodontia*). Oslo, Norway: Fungiflora. 1049-1276 p.

9. Eriksson J, Hjortstam K, Ryvarden L. (1978) The Corticiaceae of North Europe (*Mycoaciella-Phanerochaete*). Oslo, Norway: Fungiflora. 887-1048 p.

10. Eriksson J, Ryvarden L. (1976) The Corticiaceae of North Europe (*Hyphodermella-Mycoacia*). Oslo, Norway: Fungiflora. 574-886 p.

11. Eriksson J, Ryvarden L. (1975) The Corticiaceae of North Europe (*Coronicium-Hyphoderma*). Oslo, Norway: Fungiflora. 287-546 p.

12. Eriksson J, Ryvarden L. (1973) The Corticiaceae of North Europe (*Aleurodiscus-Confertobasidium*). Oslo, Norway: Fungiflora. 59-286 p.

13. Hansen L, Knudsen H, Dissing H, Ahti T, Ulvinen T, et al. (2000) Nordic macromycetes, Ascomycetes. Copenhagen, Denmark: Nordsvamp.

14. Hjortstam K, Larsson H, Ryvarden L. (1988) The Corticiaceae of North Europe (*Thanatephorus-Ypsilonidium*). Oslo, Norway: Fungiflora. 1450-1631 p.

15. Hjortstam K, Larsson H, Ryvarden L. (1987) The Corticiaceae of North Europe (introduction and keys). Oslo, Norway: Fungiflora. 59 p.

16. Jülich W, Stalpers JA. (1980) The resupinate non-poroid Aphyllophorales of the temperate northern hemisphere. Amsterdam, Oxford, New York: North-Holland Publishing Company.
